# Single‐cell RNA sequencing: Inhibited Notch2 signalling underlying the increased lens fibre cells differentiation in high myopia

**DOI:** 10.1111/cpr.13412

**Published:** 2023-01-30

**Authors:** Yunqian Yao, Ling Wei, Zhenhua Chen, Hao Li, Jiao Qi, Qingfeng Wu, Xingtao Zhou, Yi Lu, Xiangjia Zhu

**Affiliations:** ^1^ Eye Institute and Department of Ophthalmology Eye & ENT Hospital, Fudan University Shanghai China; ^2^ National Health Center Key Laboratory of Myopia (Fudan University), Key Laboratory of Myopia Chinese Academy of Medical Sciences Shanghai China; ^3^ Shanghai Research Center of Ophthalmology and Optometry Shanghai China; ^4^ Shanghai Key Laboratory of Visual Impairment and Restoration Shanghai China; ^5^ State Key Laboratory of Molecular Development Biology Institute of Genetics and Developmental Biology, Chinese Academy of Sciences Beijing China; ^6^ University of Chinese Academy of Sciences Beijing China; ^7^ Center for Excellence in Brain Science and Intelligence Technology Chinese Academy of Sciences Beijing China; ^8^ Chinese Institute for Brain Research Beijing China; ^9^ Beijing Children's Hospital Capital Medical University Beijing China; ^10^ State Key Laboratory of Medical Neurobiology Fudan University Shanghai China

## Abstract

High myopia is the leading cause of blindness worldwide. It promotes the overgrowth of lens, which is an important component of ocular refractive system, and increases the risks of lens surgery. While postnatal growth of lens is based on the addition of lens fibre cells (LFCs) supplemented by proliferation and differentiation of lens epithelial cells (LECs), it remains unknown how these cellular processes change in highly myopic eyes and what signalling pathways may be involved. Single‐cell RNA sequencing was performed and a total of 50,375 single cells isolated from the lens epithelium of mouse highly myopic and control eyes were analysed to uncover their underlying transcriptome atlas. The proportion of LFCs was significantly higher in highly myopic eyes. Meanwhile, *Notch2* signalling was inhibited during lineage differentiation trajectory towards LFCs, while *Notch2* predominant LEC cluster was significantly reduced in highly myopic eyes. In consistence, *Notch2* was the top down‐regulated gene identified in highly myopic lens epithelium. Further validation experiments confirmed NOTCH2 downregulation in the lens epithelium of human and mouse highly myopic eyes. In addition, *NOTCH2* knockdown in primary human and mouse LECs resulted in enhanced differentiation towards LFCs accompanied by up‐regulation of MAF and CDKN1C. These findings indicated an essential role of NOTCH2 inhibition in lens overgrowth of highly myopic eyes, suggesting a therapeutic target for future interventions.

## Graphical abstract

The lens epithelium from mouse highly myopic and control eyes were obtained and prepared for single‐cell RNA sequencing. In highly myopic eyes, *NOTCH2* signalling inhibition may relieve the transcriptional repression of HES1 on MAF and CDKN1C. MAF activation facilitates accumulation of β/γ crystallins which are specific structural proteins of lens fibre cells, while CDKN1C activation initiates differentiation. They together accelerate secondary lens fibre cell differentiation in highly myopic eyes.

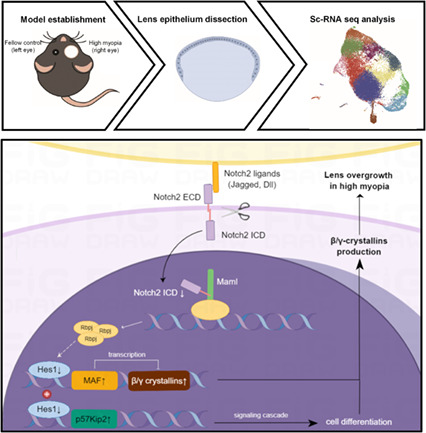

## 1 INTRODUCTION

High myopia, defined as eye axial length larger than 26 mm or spherical equivalent greater than −6.00 D, is now a leading cause of blindness and becomes increasingly prevalent across the world.^1^ Lens is an important component of ocular refractive system, and changes in its transparency or size would lead to vision impairment. We previously identified enlarged lens sizes in human highly myopic eyes associated with excessive buildup of lens structural proteins (β/γ‐crystallin),^2^, ^3^ which was recapitulated in two independent highly myopic mouse models. The enlarged lens size in high myopia contributes to higher incidences of surgical complications, such as intraocular lens malposition after lens replacement surgery, impairing visual outcomes.^3^, ^4^, ^5^ Though postnatal growth of lens is achieved through addition of fibre cells supplemented by the lifelong proliferation and differentiation of lens epithelial cells (LECs),^2^, ^6^, ^7^ it remains largely unknown about how these cellular processes get changed and play roles in highly myopic eyes.

LECs are the single layer of cells that locate underneath the anterior and equatorial part of the lens capsule. LECs underwent heterogeneous cellular processes under the same temporal dimension, including proliferation, migration, and fibre cells differentiation.^8^ Meanwhile, since they are the outermost layer of cells in the lens to get exposed to the ocular environment, LECs play essential roles for lens biology and pathology. However, there currently does not exist a characterization of transcriptional landscape involved in the heterogeneous lens epithelium biology at single‐cell levels, not to mention that how it would change in the pathological environment of highly myopic eyes and consequently induce lens abnormalities. Recently developed single‐cell RNA sequencing (sc‐RNA seq) technology now provides a potent tool for characterization of the underlying mechanism at single‐cell resolution.

In this study, we obtained mouse lens epithelium from both highly myopic and contralateral control eyes for sc‐RNA seq analysis. We outlined the single‐cell transcriptome atlas and the lineage differentiation trajectory for LECs, and found increased differentiation towards fibre cells in highly myopic eyes which was regulated by *Notch2* down‐regulation, providing a novel perspective for understanding lens abnormalities, especially lens overgrowth, in highly myopic eyes.

## 2 MATERIALS AND METHODS

### 2.1 Ethics statement

This study was affiliated to Shanghai High Myopia Study and authorized by the Eye and Ear, Nose, Throat (EENT) Hospital of Fudan University. Prior to participation, each patient provided written informed consents for collection of their clinical information and lens epithelial samples. The principles of the Declaration of Helsinki were followed in all procedures. All animal experiments were conducted according to the ARVO Statement of Animals in Ophthalmic and Vision Research with approval from the Ethics Committee for Animal Studies of Eye & ENT Hospital of Fudan University.

### 2.2 Animals

During the experiment, mice were housed in specific pathogen free (SPF) barrier facilities at a constant temperature of 21°C with 40%–60% humidity and under a regular 12 h light/dark (L/D) cycle (light on at 7:00 AM and off at 7:00 AM).

The defocus‐induced high myopia mouse model was established by attaching a −25.00 D lens to the skin around the right eye of a 4‐week‐old male C57BL/6J mouse, while the fellow eye served as control. The SLAC Laboratory Animal Co. Ltd. (China) supplied all the mice used in this study. The refraction of both eyes was measured with a mouse infrared photorefractor (Steinbeis Transfer Center, Germany) at the beginning and end of the study. Mice with baseline refraction of more than 1.00 D between eyes were excluded. The lenses were checked every morning to ensure their attachment. Four weeks later, only mice with the right eye exhibiting more than −6.00 D of myopia compared to the left eye were regarded as effective models of high myopia.

### 2.3 Lens epithelial cell isolation and single‐cell RNA sequencing

Lens epithelium (*n* = 90 for highly myopic eyes, *n* = 90 for contralateral control eyes) were isolated in DMEM (Thermo Fisher Scientific, USA) on ice. After digestion in TrypLE (Thermo Fisher Scientific) at 4°C for 4 h and then at 37°C for 15 min, cells were suspended and filtered through a 40 μm cell strainer (Bel‐Art, USA), centrifuged (300 g, 5 min), and then resuspended in PBS containing 0.04% BSA (Sigma Aldrich, USA). Using a Countess® II Automated Cell Counter (Thermo Fisher Scientific), the concentration and vitality of cells were assessed after staining with trypan blue.

Then, single cell suspensions were loaded on the Chromium Single Cell Controller (10× Genomics, USA) to generate single‐cell gel bead‐in‐emulsions (GEMs) and scRNA‐seq libraries were constructed using Single Cell Reagent Kit (10× Genomics) as per the manufacturer's protocol. The final amplified cDNA libraries were pooled and sequenced on NovaSeq 6000 (IL, USA).

### 2.4 Data pre‐processing and quality control

CellRanger software (version 4.0.0) was used to pre‐process raw sequencing data. After demultiplexing, fastq‐files were generated and aligned to mm10 mouse reference genome and transcriptome to produce gene versus cell expression matrixes. The expression matrixes were then processed using Seurat package (version 4.0.6) in R software (version 4.0.5). After quality control, cells with >200 genes and <2500 genes and <10% mitochondrial RNA were retained.

### 2.5 Single‐cell analysis

Using Seurat package in R software, the filtered expression matrixes were integrated, scaled, and normalized. Highly variable genes were detected and used for the principal component analysis (PCA). Cell clustering was subsequently performed and visualized with the top 10 principal components (PC) using uniform manifold approximation and projection (UMAP) at a resolution of 0.6. Then, we used well‐established marker genes to annotate each cell type based on their average expression.

### 2.6 Cluster markers identification and functional enrichment analysis

Differentially expressed genes (DEGs) or marker genes for each cluster were generated after running the ‘FindAllMarkers’ function, and filtered by PCT >25% (at least 25% of cells in either of the two populations compared express the gene), Log_2_ FC (Fold Change) >0.25 and *p* value (Bonferroni adjust) <0.05. The DAVID database (https://david.ncifcrf.gov/home.jsp) and the Metascape tool (http://metascape.org) was used for functional enrichment analysis.^9^, ^10^

### 2.7 Cell cycle and differentiation analysis and pseudotime transcriptional trajectory analysis

Based on the expression of genes related to the G2/M and S phases as well as differentiation states, the ‘CellCycleScoring’ function was used to determine the cell cycle and differentiation score for each cell before matching it to the metadata.^11^, ^12^ Then, cells are categorized into specific cell cycle and differentiational stages according to their scores.

We employed ‘Monocle2’ package (version 2.18.0) for pseudotime analysis using 400 marker genes generated from ‘differentialGeneTest’ function.^13^ RNA counts in all cells from LEC and LFC clusters were chosen as input for downstream analysis. Lineage differentiation trajectory among LEC and LFC clusters is performed using default parameters of ‘Monocle’ after DDRTree‐based dimensionality reduction and cell ordering. The proliferation state suggested the start point of the pseudotime when running the ‘orderCells’ function. Visualization was realized using the ‘plot_cell_trajectory’ function.

### 2.8 Primary human and mouse LEC culture and siRNA transfection

For primary human LEC culture, human lens epithelial samples from highly myopic eyes were placed with LECs facing upward and cultured in DMEM (20% FBS) at 37°C with 5% CO_2_. The medium was changed every second day. The same method was used for primary mouse LEC culture with lens epithelial samples obtained from 3‐week C57BL/6J mice. For Notch2 knockdown, cells were incubated with Notch2 siRNA at a final concentration of 50 nM transfected using Lipofectamine™ 3000 Reagent (Thermo Fisher Scientific, USA) for 48 h according to the manufacturer's protocol. A scrambled siRNA was used as the negative control. SiRNAs targeting human/mouse *Notch2* mRNA (#stB0007281B, #stB0007281C, #siG140804170557, #siG140804170633, RiboBio, China) and scrambled siRNAs were designed and synthesized by Guangzhou RiboBio Company. To avoid potential off‐target effects,^14^ two siRNA duplexes targeting different regions of the gene were used after verification of their knock‐down efficiency in either human or mouse LECs.

### 2.9 qPCR, western blotting, and immunohistochemistry

For qPCR, we used RNeasy Micro Kit (#74004, Qiagen, Germany) to extract total RNA from lens epithelium samples. The Nanodrop spectrophotometer (Thermo Fisher Scientific, USA) was used for RNA quantitation. mRNA was reverse transcribed using the PrimeScript™ RT Master Mix (#RR036, Takara, Japan) and then quantified using TB Green® *Premix Ex Taq*™ II (#RR820, Takara, Japan) on an ABI 7500 Analyser (Thermo Fisher Scientific).

For Western blotting, proteins were extracted from lens epithelium samples, followed by gel electrophoresis, transmembrane, blocking, and incubation with primary and secondary antibodies. Pierce Western Blotting Substrate Plus (Thermo Fisher Scientific, USA) were used for protein bands visualization. Density of protein bands was assessed with ImageJ software. Expression levels of targeted proteins are represented as their density ratios to that of the loading control (GAPDH) and used for following statistical analysis.

For immunohistochemistry, mice eyes were fixed in 4% paraformaldehyde for 12 h and embedded in paraffin. After dewaxing, endogenous peroxidase blockage with 3% H_2_O_2_ solution, antigen retrieval and blockage with 5% BSA, slides were then incubated with rabbit anti‐NOTCH2 antibody (1:100, #ZRB1830, Sigma Aldrich, USA) at 4°C overnight. Streptavidin‐Biotin‐Complex (SABC)‐HRP Kit (#P0615, Beyotime, China) and DAB substrate solution (#P0202, Beyotime, China) were then used for colour development according to the manufacturer's instructions. All these experiments were repeated for at least three times. The primer sequences and antibodies are shown in Tables S1 and S2.

### 2.10 Statistics analysis

Experimental data were presented as mean ± standard error (SD). Paired *t*‐test was used for the comparison of refraction between highly myopic and fellow control eyes. Independent‐sample *t*‐test was used for the comparison of other experimental data between two groups. Differences with *p* value <0.05 were considered to be statistically significant.

## 3 RESULTS

### 3.1 Single‐cell expression atlas and cell types of lens epithelium

**FIGURE 1:**
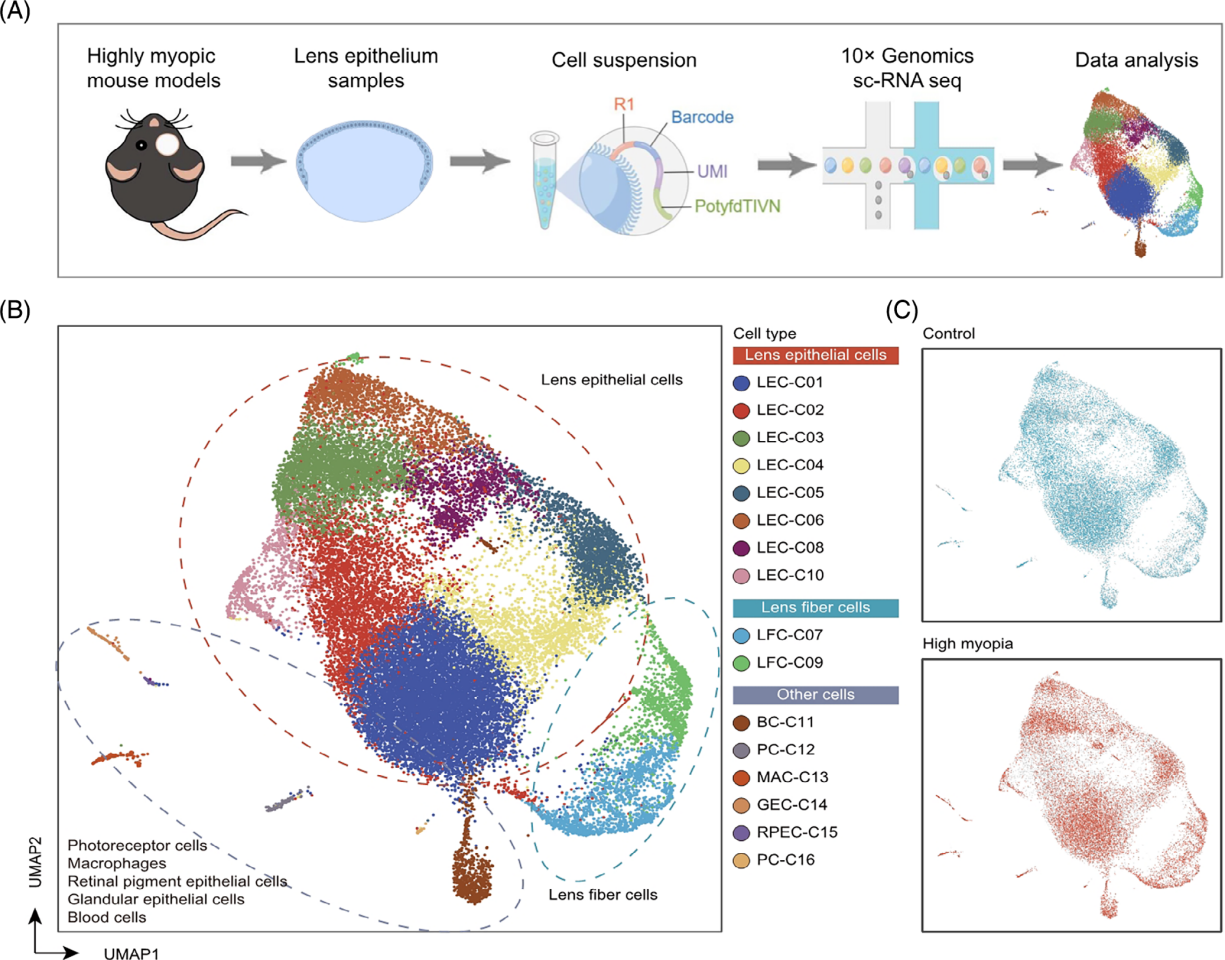
Single‐cell transcriptomic profiling of mouse lens epithelium in highly myopic and control eyes. (A) The workflow exhibiting the collection and preparing procedures of lens epithelium from mouse highly myopic and control eyes for single‐cell RNA‐sequencing. (B) UMAP visualization of all 30,001 cells isolated from the lens epithelium of highly myopic and control eyes, coloured according to cell clusters. (C) UMAP visualization of all 30,001 cells, coloured according to groups. Figure 1A was created by Figdraw (https://www.figdraw.com/static/index.html#/).

To investigate the cell profiling of lens epithelium in both highly myopic and emmetropic eyes, the defocus‐induced highly myopic mouse models were established using −25.00 D lens attached to the right eyes (Figure S1). After 4 weeks of induction, the refraction of the defocus‐induced eyes (right eyes) was significantly more myopic than that of untreated fellow eyes (left eyes) (0.62 ± 0.97 D vs. 12.89 ± 1.60 D, *p* value <0.001, paired *t*‐test). The lens epithelium was then dissected from the highly myopic and emmetropic eyes respectively, and prepared for sc‐RNA seq (Figure 1A). Using the 10× Genomics Chromium protocols, a total of 50,375 single cells are isolated. Each cell was sequenced to an average depth of 37,607 reads, resulting in a median of 807 genes and 2064 unique molecular identifiers (UMI) per cell. After filtering low‐quality cells (Figure S1), 13,787 cells from highly myopic eyes and 16,214 cells from control eyes were remained.

Based on the top 2000 highly variable genes, the clustering analysis identified a total of 16 clusters (Figure 1B,C; Data S1). A few outlier clusters highly expressed marker genes of photoreceptor cells (*Rho*, *Gngt1*),^15^ retinal pigment epithelial cells (*Lrat*, *Ttr*),^15^ glandular epithelial cells (*Muc20*, *Krt13*),^16^ macrophages (*Lyz2*, *C1qb*),^17^ and blood cells (*Hbb‐bs*, *Alas2*)^18^ (Figure S1), which represented contamination of non‐lens‐epithelium cells and were then excluded from the downstream analysis. Finally, a total of 10 clusters which highly expressed established pan‐LEC markers, such as *Cryab* (Figure S1),^14^ were identified as LECs.

**FIGURE 2:**
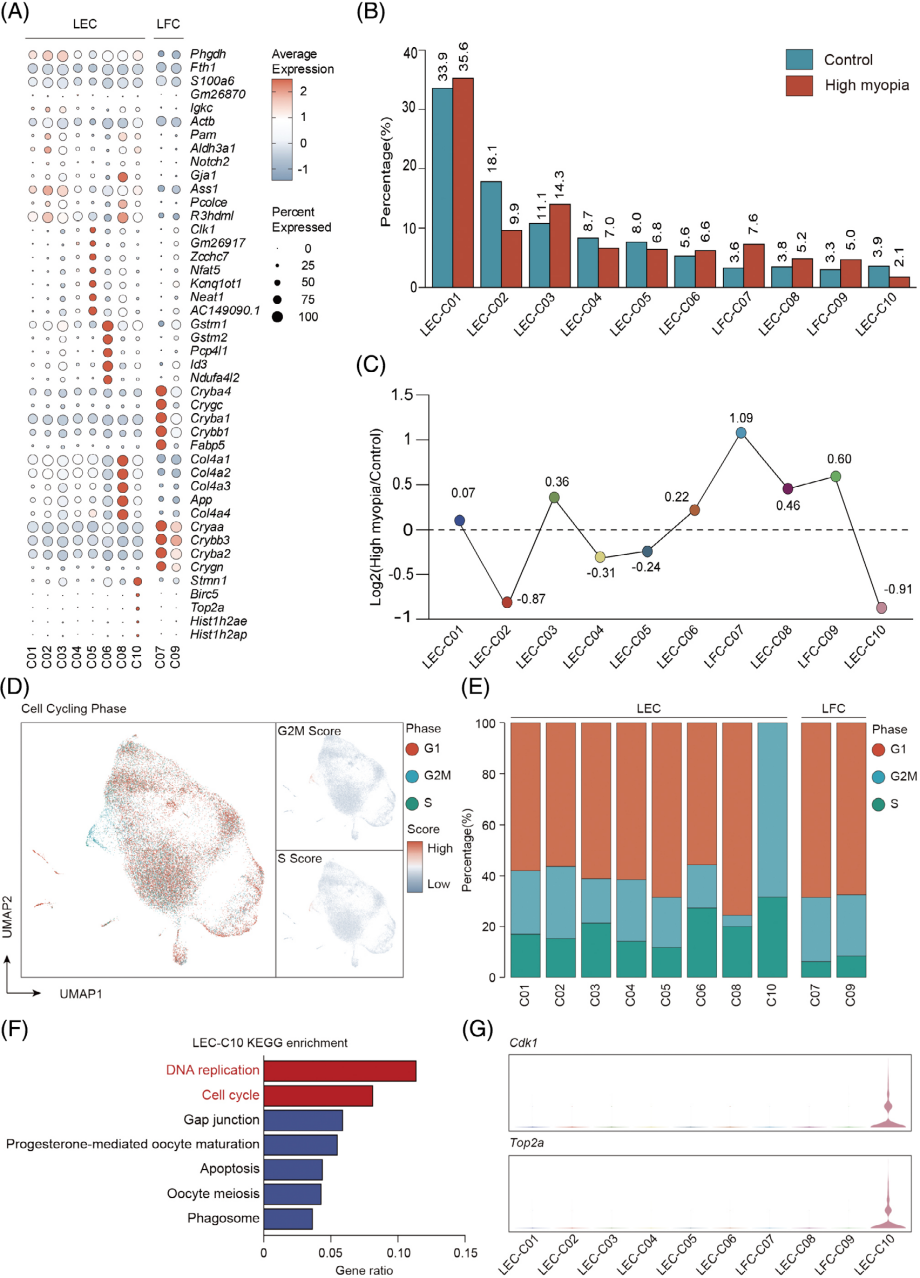
The heterogeneity of lens epithelial cells in highly myopic and control eyes. (A) Dot plot for expression of marker genes in each cell cluster of lens epithelial cells. (B) Relative proportion of each cell cluster within lens epithelial cells in highly myopic and control eyes. (C) The fold change of relative proportions of each cell cluster between highly myopic and control eyes. (D) UMAP visualization of G2M and S scores for each cell in highly myopic and control lens epithelium, calculated based on their expression levels of cell‐cycle marker genes. (E) Relative proportion of cells with different cell cycling phases within each cell cluster. (F) KEGG enrichment analysis results of the up‐regulated genes for cluster 10. (G) Violin plot showing the specific expression of cell cycle marker *Cdk1* and cell proliferation marker *Top2a* in cluster 10.

**FIGURE 3:**
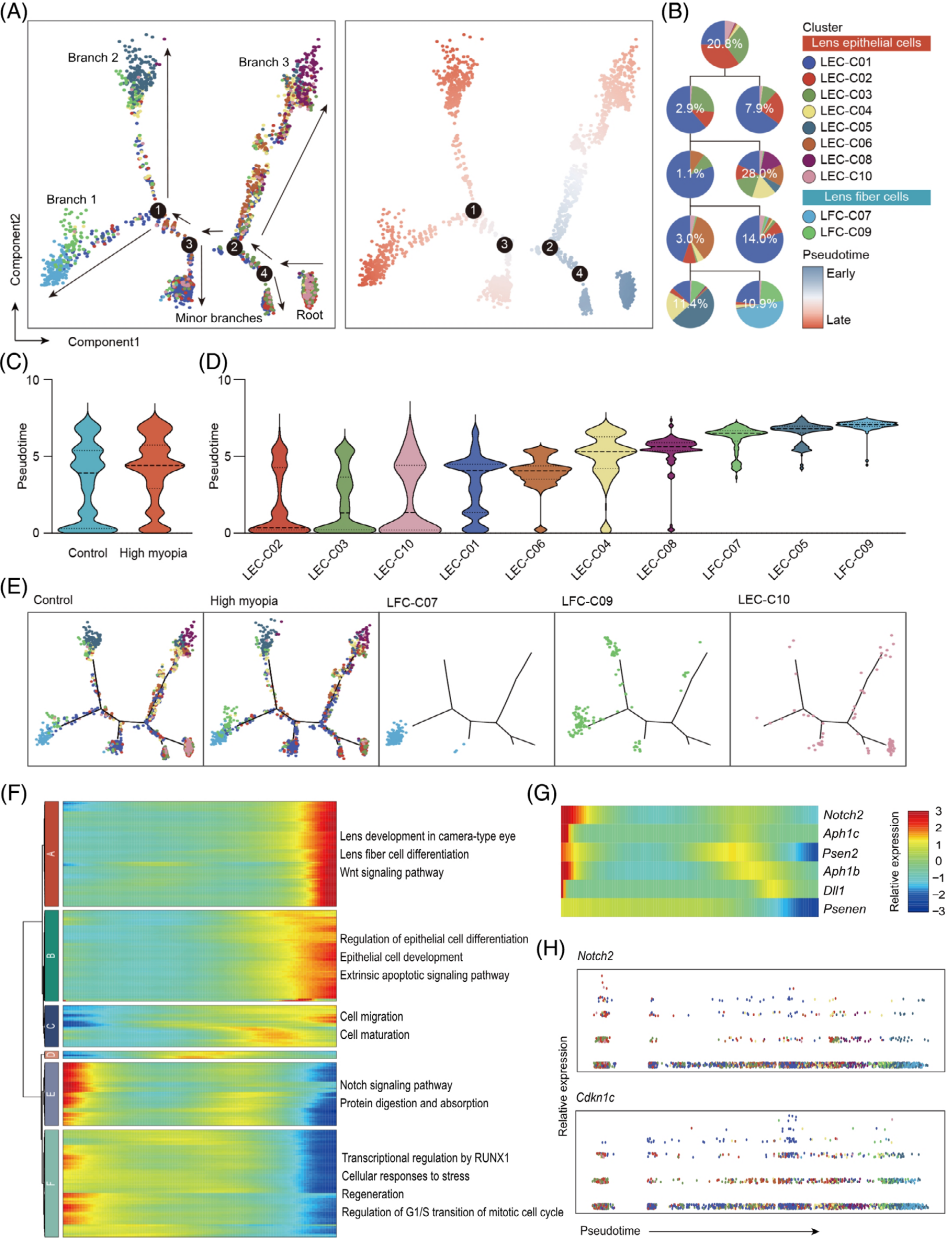
The differentiation trajectory of lens epithelial cells predicted by pseudotime analysis. (A) Pseudotime of lens epithelial cells generated by Monocle2 package of R software, coloured by cell clusters and pseudotime. Each dot represents a single cell. The pseudotime algorithm ordered the cells into one root, three major branches and two minor branches. (B) Relative proportions of cell clusters for each cell state along the differentiation trajectory. (C) Pseudotime of lens epithelial cells in highly myopic eyes and control eyes. (D) Pseudotime of cluster 7, 9, and 10. (E) Violin plot displaying the calculated pseudotime of lens epithelial cells in highly myopic and control eyes and each cell cluster. (F) The representative gene functions and pathways of each profile enriched from the differentially expressed genes along the pseudotime trajectory. (G) Dynamic expression profiles of Notch signalling, especially *Notch2* expression, along pseudotime differentiation trajectory. (H) Dynamic expression profiles of *Notch2* and *Cdkn1c* along pseudotime trajectory.

By comparing gene expression patterns, we found that these cell clusters can be distinguished by specific gene sets (Figure 2A). The proportions of each LEC cluster in highly myopic and control lens epithelium were exhibited in Figure 2B,C. We firstly scored each cell based on their expression levels of G2M or S phase marker genes. We found that all the cells in cluster 10 exhibited high G2M or S scores, and were therefore classified to be in either G2M or S phase of cell cycle, whereas the majority of cells in other clusters were identified to be in the G1 phase (Figure 2D,E). Cluster 10 was then identified as a proliferating LEC subpopulation, as confirmed by its enriched function for cell proliferation and specific expression of relevant markers (e.g., *Top2a* and *Cdk1*) (Figure 3F,G). Since LECs undergo distinct cellular states under natural conditions, functional enrichment analysis was performed for each cluster using their marker genes (Figure S2). Roles of genes specific for cluster 1, 3, 4, and 6 were related to regulation of cell morphogenesis (e.g., *Anxa1*, *S100a10*, and *Myl12a*), collagen formation (e.g., *Ctsb*, *Ctsl*, *Pcolce*, and *Ppib*), cell junction organization (e.g., *Cdh2*, *Hdac7*, and *Gjd3*), and extracellular matrix organization (e.g., *Ccn2*, *Nid1*, and *Prdx4*), respectively, indicating that they performed basic functions of maintaining cell polarity and constituting lens capsular matrix. Genes of cluster 2 was enriched for regulation of cell population proliferative and developmental process (e.g., *Notch2*, *Ctsl*, *Gja1*, and *Ecrg4*), suggesting its possible regulative effects on lens growth and development. Cluster 8 specifically expressed type IV collagen genes (*Col4a1*, *Col4a2*, *Col4a3*, and *Col4a4*) (Figure 2A), and was enriched for epithelial cell migration (e.g., *Efnb2*, *Itgb1*, and *Slit2*), suggesting that there existed a proportion of LECs related to epithelial‐mesenchymal transition, cell migration and lens fibrotic diseases.^19^, ^20^ Remarkably, cluster 7 and 9 highly expressed crystallin β/γ genes (e.g., *Cryba2*, *Crybb1*, and *Crygn*) (Figure 2A) and could therefore be further annotated as fibre cells as a result of LEC differentiation.^21^ On the contrary, genes for cluster 5 were enriched for negative regulation of epithelial cell differentiation (e.g., *Notch1*, *Hes1*, *Tgfbr1*, and *Vegfa*) and homeostasis maintenance of cell numbers within the tissue.

### 3.2 Pseudotime analysis exhibited differentiation trajectory of lens epithelial cells

To characterize the differentiation trajectory of LECs, pseudotime analysis was further conducted, where cells were gathered along a trajectory according to their gene signatures and calculated pseudotime without prior clustering information (Figure 3A). Cluster 1 was presented throughout the pseudotime trajectory and composed the majority of 2 minor branches, indicating that maintaining the morphogenesis of polarized epithelium was needed during all these processes. Except for that, the cells were ordered into 1 root and 3 major branches by the unsupervised algorithm. Mitotic and post‐mitotic LECs (cluster 10 and cluster 2) characteristically populated at the root of the trajectory, while the three major branches were mostly composed of maturely differentiated LFCs (branch 1; cluster 7, and 9), homeostasis‐maintaining cells (branch 2; cluster 5) and static or migrating cells performing basic functions (branch 3; cluster 3, 4, 6, and 8), respectively. These results were consistent with the expression atlas defined by UMAP, further supporting our prior observations. Figure 3C exhibited the average pseudotime of all cells in highly myopic and control lens epithelium, and Figure 3D exhibited that of each LEC cluster. In consistence with functional enrichment analysis, proliferating population (cluster 10) appeared at early periods, while differentiating population (cluster 7 and 9) appeared at terminal periods (Figure 3D, E). We further analysed the dynamic gene expression patterns along the trajectory of LEC differentiation. Of note, genes enriched for Notch signalling pathway (*Notch2*, *Psenen*, *Psen2*, *Aph1b*, *Aph1c*, and *Dll1*) were found to be significantly inhibited during lineage differentiation trajectory of LECs (Figure 3F). The expression patterns of all genes related to Notch signalling pathway in each LEC cluster was exhibited in Figure S3. Of these genes, *Notch2* showed the highest level of association with pseudotime (q value = 4.96 × 10^−41^) and remarkably decreased at early periods (Figure 3G,H), indicating its potential role in regulating the differential process of LECs.

### 3.3 Higher proportion of differentiated fibre cells in lens epithelium of highly myopic eyes

**FIGURE 4:**
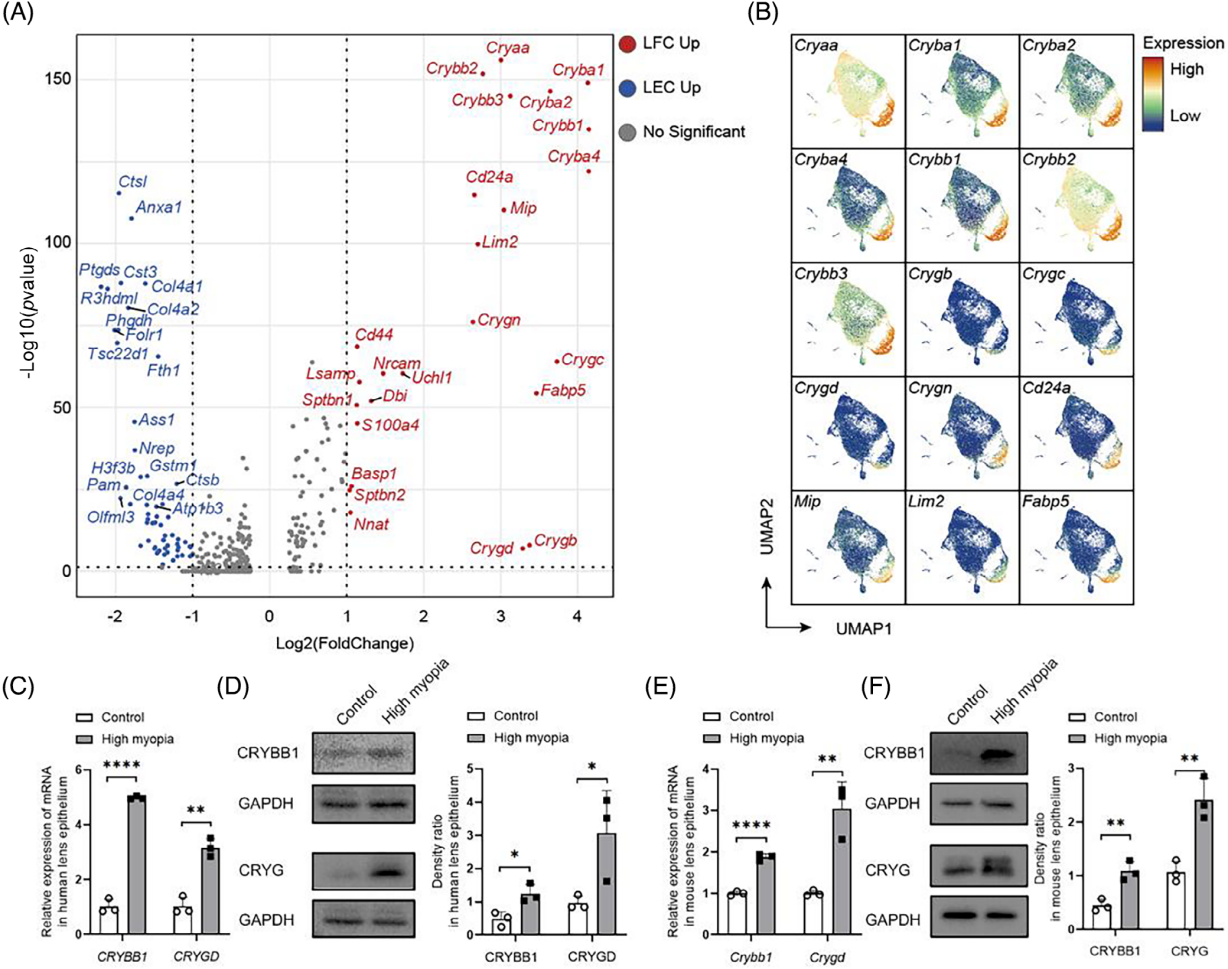
Increased differentiation towards lens fibre cells in highly myopic lens epithelium. (A) Volcano plot displaying differentially expressed genes (DEGs) detected between fibre cells (cluster 7 and 9) and undifferentiated lens epithelial cells (cluster 1–6, 8, and 10). (B) UMAP visualization of all cells isolated from lens epithelium, coloured according to the expression levels of top 15 up‐regulated DEGs of fibre cells compared to all other cell clusters. (C) Up‐regulated mRNA levels of *CRYBB1* and *CRYGD* (2 of the lens fibre cell marker genes) in human highly myopic lens epithelium. (D) Up‐regulated protein levels of CRYBB1 and CRYG in human highly myopic lens epithelium. (E) Up‐regulated mRNA levels of *Crybb1* and *Crygd* in mouse highly myopic lens epithelium. (F) Up‐regulated protein levels of CRYBB1 and CRYG in mouse highly myopic lens epithelium.

As was shown in Figure 2C, the proportion of LFCs (cluster 7 and 9) was significantly higher in highly myopic eyes. In consistence, the average pseudotime of LECs in highly myopic eyes was higher than that in control eyes (Figure 3C), which further confirmed an increased cell‐state transitions towards terminal differentiation in these eyes. Differential gene expression analyses discovered known and novel markers of LFCs compared to LECs (Figure 4A). Among the top 15 marker genes of LFCs, top 11 were all crystallin genes, and 10 of 11 were β/γ‐crystallins (Figure 4B). Besides, LFCs are also distinguishable from LECs with *Cd24a*, *Mip*, *Lim2*, and *Fabp5* (Figure 4B). While previous investigations have confirmed the specific expressive patterns of crystallins, *Mip* and *Lim2* in LFCs,^22^, ^23^ *Cd24a* and *Fabp5* are newly‐identified LFC markers which were reported to get involved in differentiation in other cell lineages.^24^, ^25^ On the other hand, the proliferating population (cluster 10) was lower in highly myopic eyes (Figure 2C), suggesting an accelerated transition from proliferation to differentiation. Using qPCR and Western blotting, we confirmed the up‐regulated mRNA and protein levels of fibre cell markers (CRYBB1, CRYG) in lens epithelium dissected from human highly myopic eyes compared to those of control eyes (Figure 4C,D). Up‐regulation of CRYBB1 and CRYG was further validated in lens epithelium samples from mouse highly myopic eyes (Figure 4E,F).

### 3.4 Down‐regulation of Notch2 promoted differentiation towards fibre cells in the lens epithelium of highly myopic eyes

**FIGURE 5:**
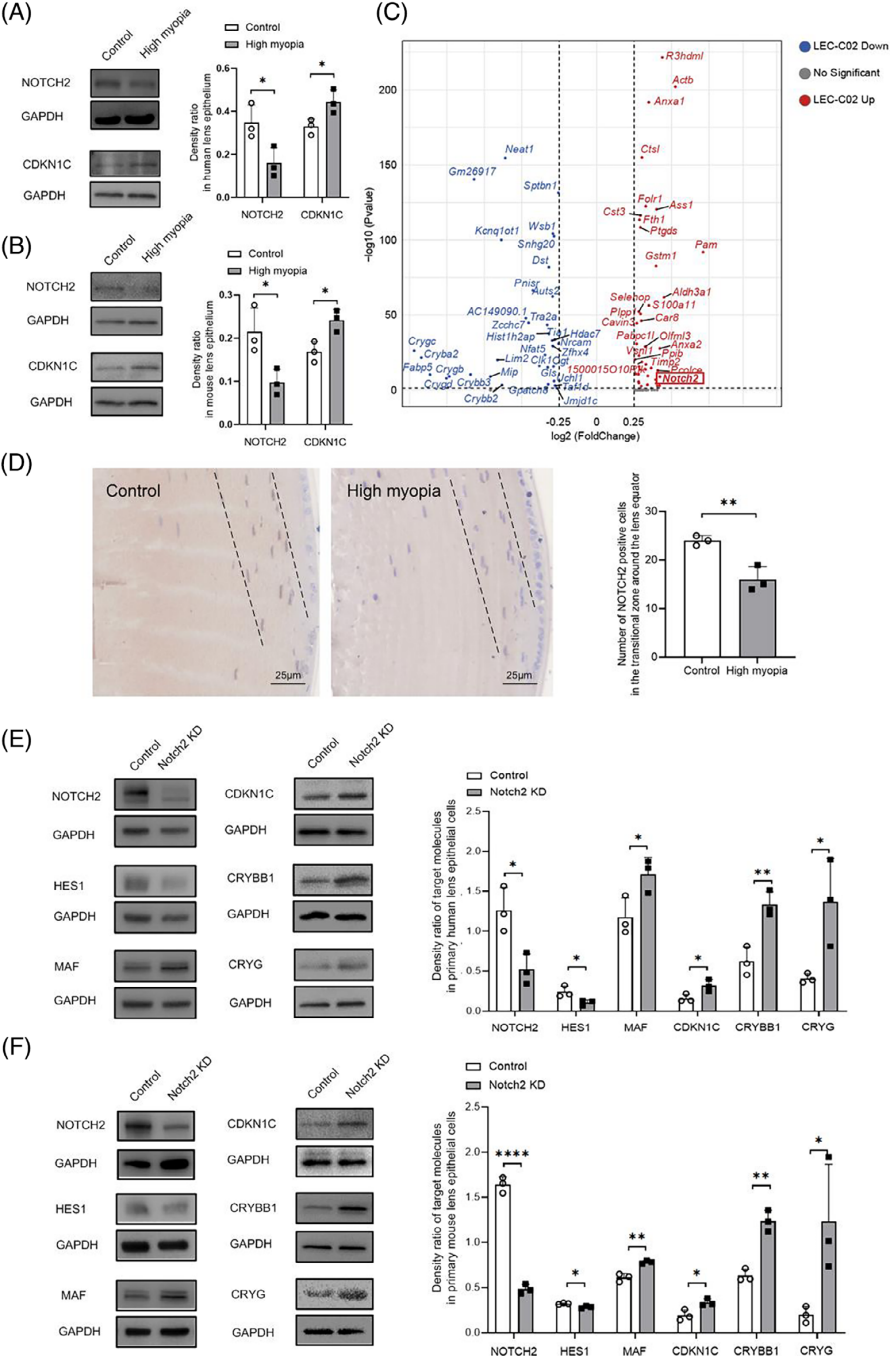
Notch2 signalling inhibition promotes differentiation towards lens fibre cells in highly myopic lens epithelium. (A) Down‐regulated protein level of NOTCH2 in human highly myopic lens epithelium. (B) Down‐regulated protein level of NOTCH2 in mouse highly myopic lens epithelium. (C) Volcano plot displaying differentially expressed genes detected between cluster 2 and any other LEC cluster. *Notch2* was significantly activated in cluster 2 compared with other LEC clusters (Log_2_ FC = 0.42, adjusted *p* value = 1.29 × 10^−9^). (D) Immunohistochemistry for *NOTCH2* showing the distribution of NOTCH2‐expressing cells in the transitional zone (the area between two dotted lines) around the lens equator and a significant reduction in the number of NOTCH2‐expressing cells in highly myopic lens. (E) Western blotting analysis of downstream molecules in primary cultured human lens epithelial cells in response to *NOTCH2* knockdown with siRNA. (F) Western blotting analysis of downstream molecules in primary cultured mouse lens epithelial cells in response to *Notch2* knockdown with siRNA. Following *NOTCH2* knockdown, its effector HES1 was significantly down‐regulated, while MAF, CDKN1C and fibre cell markers (CRYBB1 and CRYG) were significantly up‐regulated in both human and mouse primary lens epithelial cells. Data are expressed as mean ± SEM. *p < 0.05; **p < 0.01; ***p < 0.001; ****p < 0.0001; Student's *t*‐test.

To determine the possible mechanism underlying the increased fibre cells in the lens epithelium of highly myopic eyes, we combined the results from pseudotime analysis and biological functions underlying significantly altered clusters in these eyes. Prior pseudotime analysis revealed the down‐regulation of *Notch* signalling along lens differentiation trajectory. Considering that predominant changes could exhibit at the tissue level, we performed qPCR to confirm the specific molecules that have the greatest potential to get involved in the LEC differentiation process of highly myopic eyes. While the mRNA levels of *Psenen*, *Psen2*, *Aph1b*, *Aph1c*, and *Dll1* exhibited no significant differences (Figure S4), mRNA and protein levels of NOTCH2 were both remarkably down‐regulated in lens epithelium dissected from both human and mouse highly myopic eyes compared to that of control eyes (Figure 5A,B). This result was consistent with differential gene expression analysis that *Notch2* was the top down‐regulated gene identified in highly myopic lens epithelium compared with control eyes (log_2_FC = −0.26, adjusted *p* value = 2.75 × 10^−296^) (Figure **S5**A).

On the other hand, as was shown in Figure 2C, the proportion of cluster 2 was significantly lower in highly myopic eyes, indicating its vulnerability to high myopia. *Notch2* was significantly activated in cluster 2 compared with other LEC clusters (Figure 5C). Therefore, immunohistochemistry was performed to confirm the distribution of these NOTCH2‐expressing cells. As was shown in Figure 5D, activated NOTCH2 signalling spatially specifically distributed in the transitional zone around the lens equator, where post‐mitotic LECs are undergoing fate determination and prepared to differentiate into LFCs.^26^ Of note, consistent with the significant reduction in the cluster 2 proportion, NOTCH2‐expressing cells were significantly reduced in highly myopic lens epithelium (Figure 5D). The expression level of NOTCH2 signalling was also remarkably down‐regulated in these cells of highly myopic eyes. These results indicate that NOTCH2 signalling inhibition may accelerate the process towards differentiation of these NOTCH2‐expressing cells, therefore participating in promoting fibre cell differentiation in highly myopic eyes.

Next, we assessed the biological role of *Notch2* on fibre cell differentiation. As the immortalized cell line lacked the ability to differentiate, human and mouse primary lens epithelial cells were used. We found that *NOTCH2* inhibition by siRNA increased the mRNA levels (Figure S5) and protein levels (Figure 5E,F) of fibre cell markers in both human and mouse primary LECs, indicating that *NOTCH2* could inhibit secondary lens fibre differentiation. Furthermore, the downstream NOTCH effector HES1 was simultaneously down‐regulated following *NOTCH2* inhibition (Figure 5E,F), while other known downstream molecules including *Hes5*, *Hey1*, and *Hey2* were not significantly altered (Figure S5). Meanwhile, the transcription factor MAF and cyclin‐dependent kinase inhibitor CDKN1C was significantly up‐regulated (Figure 5D,E, Figure S5). These results were further confirmed by repeated transfection experiments using a different siRNA molecule targeting a different region of *NOTCH2* (Figure S5).

## 4 DISCUSSION

High myopia is a blinding eye disease with high prevalence across the globe. It was estimated to affect nearly 1 billion people by 2050.^1^ The extensive elongation of eyeballs dramatically increases the risk of a variety of ocular pathologies, including lens diseases such as early‐onset cataract and lens dislocation,^27^, ^28^ resulting in poor visual prognosis. Our previous studies further demonstrated a pathologically increased lens size in highly myopic eyes which may account for their higher IOL malposition incidence.^2^ Here, using a sc‐RNA seq strategy, we uncovered the gene‐expression changes associated with the increased LECs differentiation towards fibre cells in highly myopic lens epithelium. Our data also demonstrated that the NOTCH2 signalling pathway plays an essential role in LEC fate determination and lens size control even in adulthood, and its inhibition participates in increased fibre cell differentiation in highly myopic eyes.

We previously verified the pathological growth of lens in both human and mouse highly myopic eyes using magnetic resonance imaging (MRI).^2^ It indicated that high myopia is not merely associated with the elongation of eyeballs; the morphology of lens would change accordingly. Lens epithelium, a simple cuboidal epithelium, locates between lens fibres and lens capsule, with critical functions in providing progenitors for continuous lens growth throughout the life‐span.^29^, ^30^ While traditional bulk‐RNA sequencing approaches could not resolve the process of cellular phenotype shift, in this study, we used sc‐RNA seq methodology to define the phenotype and functional heterogeneity of LEC subpopulations.

This analysis initially uncovered 10 functionally distinct LEC clusters, which could further be classified into two subpopulations with distinct gene expression profiles and showing significant difference between highly myopic and control eyes. Particularly, the proportion of differentiating fibre cells increased in lens epithelium of highly myopic eyes, characterized by their specific expression of β/γ‐crystallins.^21^, ^31^ β‐ and γ‐crystallins are among the three categories of crystallins which together make up about 90% of lens structural proteins,^32^ and these two families account for more than 77% of all crystallins.^2^, ^32^ They are expressed specifically in LFCs, and therefore serve as LFC molecular markers.^21^, ^31^ This is consistent with a previous bulk‐RNA sequencing result showing up‐regulation of β/γ‐crystallins in highly myopic lens epithelium,^2^ and has been confirmed through our validation experiments. The accumulation of differentiated fibre cells therefore provides a structural foundation for increased lens size in highly myopic eyes.

In the current study, we utilized an unsupervised method to outline the pseudotime trajectory of LEC differentiation and visualize the dynamic alternations of genes of interest. We found that the NOTCH signalling pathway was significantly inhibited during differentiation towards secondary fibre cells. Particularly, NOTCH2 rather than other NOTCH receptor subtypes exhibited predominant down‐regulation. Moreover, we found that the proportion of the second largest LEC subcluster (cluster 2), characterized by high expression of *Notch2*, was significantly decreased in adult mouse highly myopic eyes. Considering their specific spatial distribution within the transitional zone between lens progenitor cells and mature LFCs, these results suggest that these NOTCH2‐expressing cells have just gone through mitosis and are undergoing cell fate determination towards LFC differentiation, and *Notch2* signalling inhibition may participate in promoting lens fibre cell differentiation in adult highly myopic eyes.

Canonical NOTCH pathway functions via direct cell–cell communication between NOTCH receptors (NOTCH1, NOTCH2, NOTCH3, and NOTCH4) and associated ligands.^33^ The NOTCH receptor extracellular domain (NECD) will be cleaved during ligand activation, releasing the NOTCH intracellular domain (NICD) which moves into the nucleus and releases the DNA‐binding protein (RBPJ). The transcription complex formed by RBPJ and the recombinant signal‐binding protein then causes the induction of NOTCH target genes, such as transcription repressors of the HES family.

NOTCH signalling engages in the determination of cell fates in a broad variety of cell types. It generally promotes progenitor or stem cell proliferation and prevents differentiation.^33^, ^34^, ^35^, ^36^, ^37^, ^38^, ^39^ Multiple lines of evidence have indicated NOTCH signalling as a key modulator of normal lens development in embryonic and fetal mice. Jia et al. and Rowan et al. both found an accelerated differentiation of primary lens fibre cells in *Rbpj*‐lens‐conditionally‐knockout (CKO) mice.^7^, ^40^ However, since NOTCH receptors have four subtypes, these two studies cannot answer the question of which individual subtype being required for such a regulatory effect. Using *Notch2*‐CKO mice, Saravanamuthu et al. found that removal of *Notch2* also resulted in an increased proportion of fibre cells, as was found in *Rbpj* mutant lenses,^41^ yet very few studies have investigated its ability to regulate secondary fibre cell differentiation in adult vertebrates.

In this study, we revealed a significantly decreased expression of NOTCH2 in the lens of human and mouse highly myopic eyes, confirming the results identified from sc‐RNA seq analysis. To validate the specific role of NOTCH2, it was inhibited using *NOTCH2* siRNA, following which its effector molecule HES1 was down‐regulated, while MAF, CDKN1C and fibre cell markers were up‐regulated. These experiment results indicated that NOTCH2 affects LEC differentiation, and MAF and CDKN1C are possible mediators. MAF is a transcription factor which has been reported to control embryonic lens development.^42^ It remains activated after birth and could regulate postnatal lens growth by directly interacting with promoters of β/γ‐crystallin genes and activating their expressions.^2^ Similar to our findings, Eom identified increased expression of MAF in the mouse pancreas resulting from *Notch* downregulation.^43^ CDKN1C is a cell cycle‐dependent kinase inhibitor that promotes withdrawal from cell cycle and initiating differentiation.^44^ Previous investigations reported CDKN1C activation in the lens of *Notch2* or *Rbpj* CKO mice.^7^, ^45^ Meanwhile, CDKN1C have been shown to be directly regulated by *Notch2* downstream transcriptional factor HES1 in pancreas and intestine.^45^, ^46^

**FIGURE 6:**
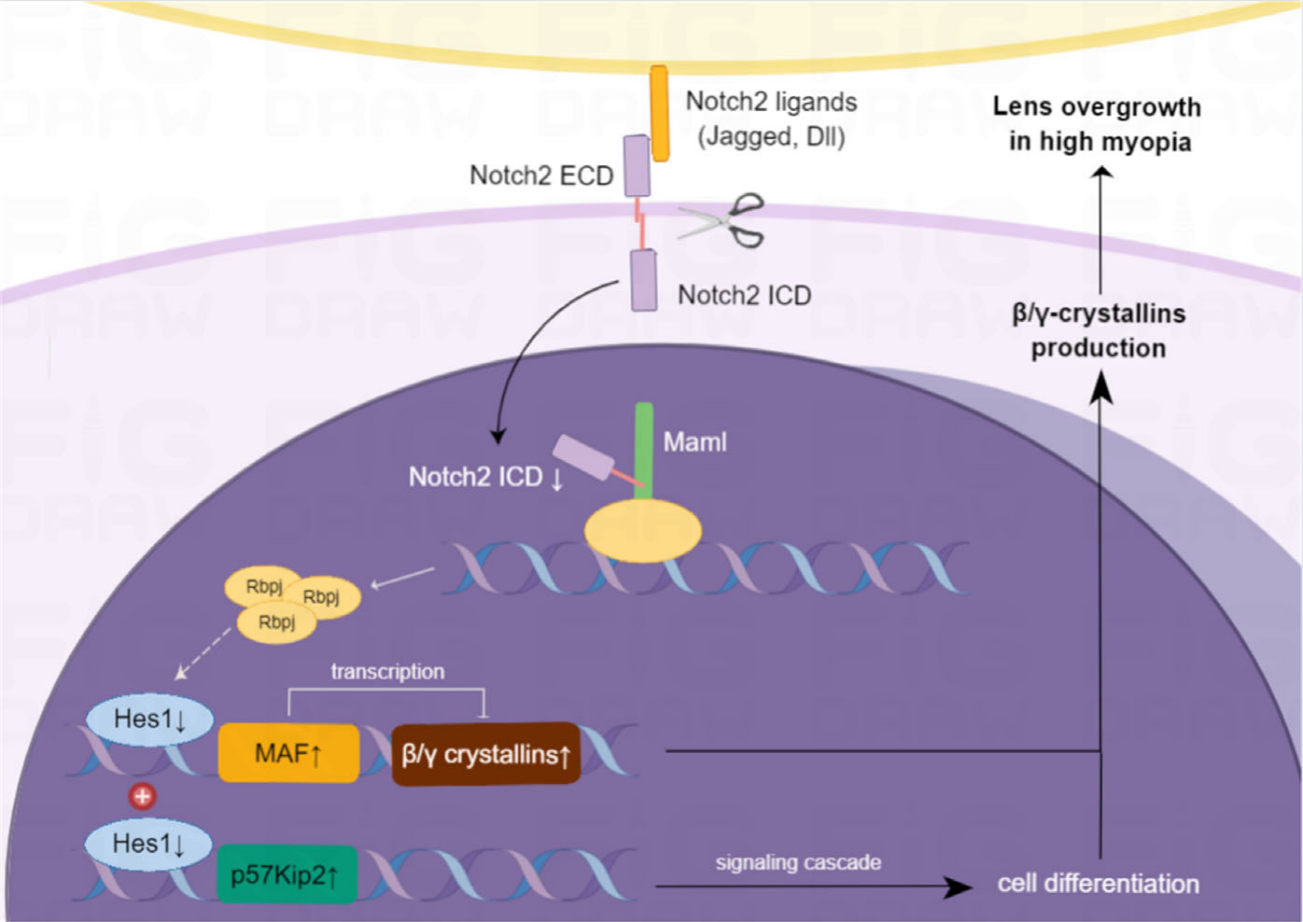
Schematic illustration of NOTCH2 inhibition underlying the increased differentiation towards lens fibre cell in highly myopic lens epithelium. In highly myopic eyes, *NOTCH2* signalling inhibition may relieve the transcriptional repression of HES1 on MAF and CDKN1C. MAF activation facilitates accumulation of β/γ crystallins which are specific structural proteins of lens fibre cells, while CDKN1C activation initiates differentiation. They together accelerate secondary lens fibre cell differentiation in highly myopic eyes. This figure was created by Figdraw.

Taken together, we suppose that in high myopia, NOTCH2 signalling inhibition could accelerate secondary lens fibre cell differentiation by activating CDKN1C, the differentiation initiator, and promote accumulation of β/γ crystallin, the specific structural proteins of LFCs, by activating MAF (Figure 6). These mechanisms collectively contribute to the overgrowth of lens in highly myopic eyes. As the expression level of HES1 decreases following *NOTCH2* knockdown, it was speculated that its transcriptional repression on MAF and CDKN1C was relieved. Importantly, using primary LECs cultured from adult human lens epithelium, our results indicated that NOTCH2 could affect LEC differentiation and lens structure even in grown‐ups. In the future, with persistently increasing evidence, the functions of cross‐talk between these pathways in high myopia associated lens diseases worth further investigation.

In conclusion, our study uncovered the transcriptome atlas and lineage differentiation trajectory of LECs, and revealed the gene expression signatures of LEC subpopulations. We validated the role of NOTCH2 signalling inhibition underlying the increased differentiation towards fibre cells in highly myopic eyes, providing a novel perspective and potential therapeutic target for lens overgrowth in highly myopic eyes.

## AUTHOR CONTRIBUTIONS

Yunqian Yao and Ling Wei contributed equally to this work. Yunqian Yao, Ling Wei and Xiangjia Zhu conceived the idea. Yunqian Yao and Xingtao Zhou wrote the initial and subsequent revised versions of the manuscript. Xiangjia Zhu, Yi Lu, and Xingtao Zhou supervised the study and were involved in critical revision of manuscript. Qingfeng Wu provided technical and material support. Yunqian Yao and Zhenhua Chen performed bioinformatic analyses. Yunqian Yao, Ling Wei, Hao Li, and Jiao Qi performed experiments and analysed data. All authors reviewed the results and approved the final version of the manuscript.

## FUNDING INFORMATION

This article was supported by research grants from the National Natural Science Foundation of China (No. 82122017, 82271069, 81870642, 81970780, 81670835, and 81470613), Science and Technology Innovation Action Plan of Shanghai Science and Technology Commission (No. 19441900700, 21S31904900 and 20410710100), Clinical Research Plan of Shanghai Shenkang Hospital Development Center (No. SHDC2020CR4078, SHDC12019X08, SHDC12020111 and SHDC2020CR1043B), Double‐E Plan of Eye & ENT Hospital (SYA202006), Shanghai Municipal Key Clinical Specialty Program (shslczdzk01901), the Fudan University Outstanding 2025 Program, Project of Shanghai Xuhui District Science and Technology (2020‐015), Shanghai Engineering Research Center of Laser and Autostereoscopic 3D for Vision Care (20DZ225500), and Construction of a 3D digital intelligent prevention and control platform for the whole life cycle of highly myopic patients in the Yangtze River Delta (21002411600).

## CONFLICT OF INTEREST STATEMENT

None.

## Supporting information


**Data S1.** Supporting Information

**Supplementary Figure S1.** (A) A representative photo of the defocus‐induced high myopia mouse model wearing a −25.00 D lens onto the right eye, while the fellow eye served as control. (B) Violin plots showing the number of genes per cell (nFeature_RNA), UMIs per cell (nCount_RNA) and the proportion of mitochondrial genes (percent.mt) in high myopia and control groups after quality control. (C) Violin plots and feature plots showing the expression patterns of marker genes of identified non‐LECs, including photoreceptor cells (*Rho*, *Gngt1*; Cluster 12 and 16), retinal pigment epithelial cells (*Lrat*, *Ttr*; Cluster 15), glandular epithelial cells (*Muc20*, *Krt13*; Cluster 14), macrophages (*Lyz2*, *C1qb*; Cluster 13), and blood cells (*Hbb‐bs*, *Alas2*; Cluster 11). (D) Violin plot and feature plot showing the expression pattern of the pan‐LEC marker, *Cryab*.

**Supplementary Figure S2.** (A–I) Enriched gene ontology terms of marker genes in LEC cluster 1–9, respectively.

**Supplementary Figure S3.** Heat map showing the expression patterns of all genes related to Notch signalling pathway in LEC clusters.

**Supplementary Figure S4.** The mRNA levels of genes related to NOTCH signalling revealed by pseudotime analysis in human and mouse highly myopic lens epithelium. ns, not statistically significant.

**Supplementary Figure S5.** (A) Volcano plot displaying differentially expressed genes (DEGs) detected between the highly myopic and control lens epithelium. (B) The mRNA levels of downstream molecules in primary cultured human lens epithelial cells in response to *NOTCH2* knockdown with siRNA. (C) The mRNA levels of downstream molecules in primary cultured mouse lens epithelial cells in response to *Notch2* knockdown with siRNA. *p < 0.05; **p < 0.01; Student's *t*‐test. (D) Western blotting analysis of downstream molecules in primary cultured human lens epithelial cells in response to *NOTCH2* knockdown with siRNA targeting another region of *NOTCH2*. (E) Western blotting analysis of downstream molecules in primary cultured mouse lens epithelial cells in response to *Notch2* knockdown with siRNA targeting another region of *Notch2*. Following *NOTCH2* knockdown, its effector HES1 was significantly down‐regulated, while other downstream molecules, *Hes5*, *Hey1*, and *Hey2* were not significantly changed, accompanied by the significant up‐regulation of MAF, CDKN1C, and fibre cell markers (CRYBB1 and CRYG) in both human and mouse primary lens epithelial cells. Data are expressed as mean ± SEM. *p < 0.05; **p < 0.01; ***p < 0.001; ****p < 0.0001; Student's *t*‐test.

**Supplementary Table S1.** Primer sequences for qPCR.
**Supplementary Table S2.** Antibody list.

## Data Availability

I confirm that my article contains a Data Availability Statement even if no data is available (list of sample statements) unless my article type does not require one (e.g., Editorials, Corrections, Book Reviews, etc.).I confirm that I have included a citation for available data in my references section, unless my article type is exempt.
