# Supplementary figures and images for: Single‐cell RNA sequencing: Inhibited Notch2 signalling underlying the increased lens fibre cells differentiation in high myopia
Source: Cell Prolif. 2023 Jan 30;56(8):e13412. doi: 10.1111/cpr.13412 (PMC10392066; doi:10.1111/cpr.13412)

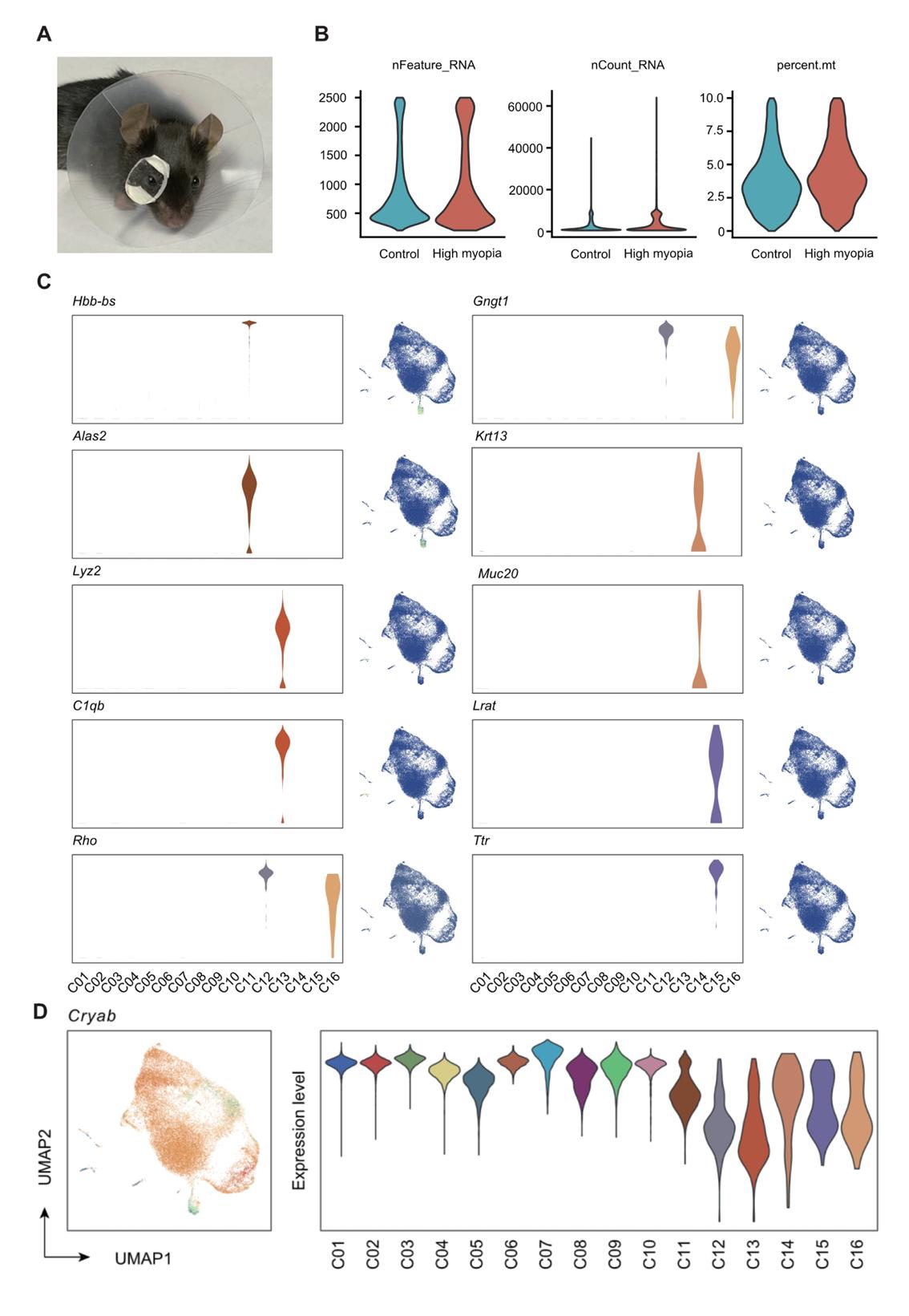

Supplement: Supplementary file 2 — Supplementary Figure S1. (A) A representative photo of the defocus‐induced high myopia mouse model wearing a −25.00 D lens onto the right eye, while the fellow eye served as control. (B) Violin plots showing the number of genes per cell (nFeature_RNA), UMIs per cell (nCount_RNA) and the proportion of mitochondrial genes (percent.mt) in high myopia and control groups after quality control. (C) Violin plots and feature plots showing the expression patterns of marker genes of identified non‐LECs, including photoreceptor cells (Rho, Gngt1; Cluster 12 and 16), retinal pigment epithelial cells (Lrat, Ttr; Cluster 15), glandular epithelial cells (Muc20, Krt13; Cluster 14), macrophages (Lyz2, C1qb; Cluster 13), and blood cells (Hbb‐bs, Alas2; Cluster 11). (D) Violin plot and feature plot showing the expression pattern of the pan‐LEC marker, Cryab. [file CPR-56-e13412-s005.jpg]

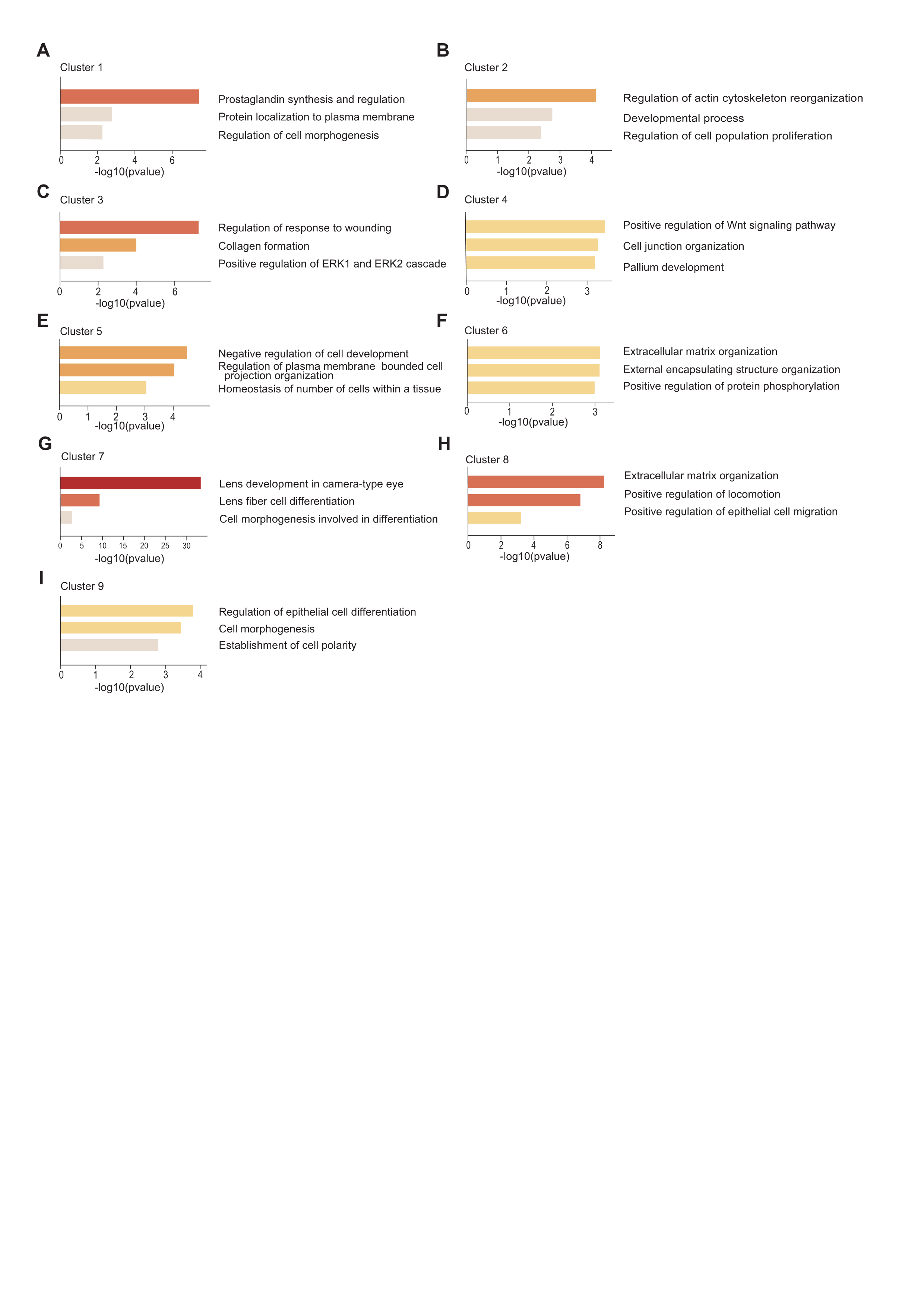

Supplement: Supplementary file 3 — Supplementary Figure S2. (A–I) Enriched gene ontology terms of marker genes in LEC cluster 1–9, respectively. [file CPR-56-e13412-s003.tif]

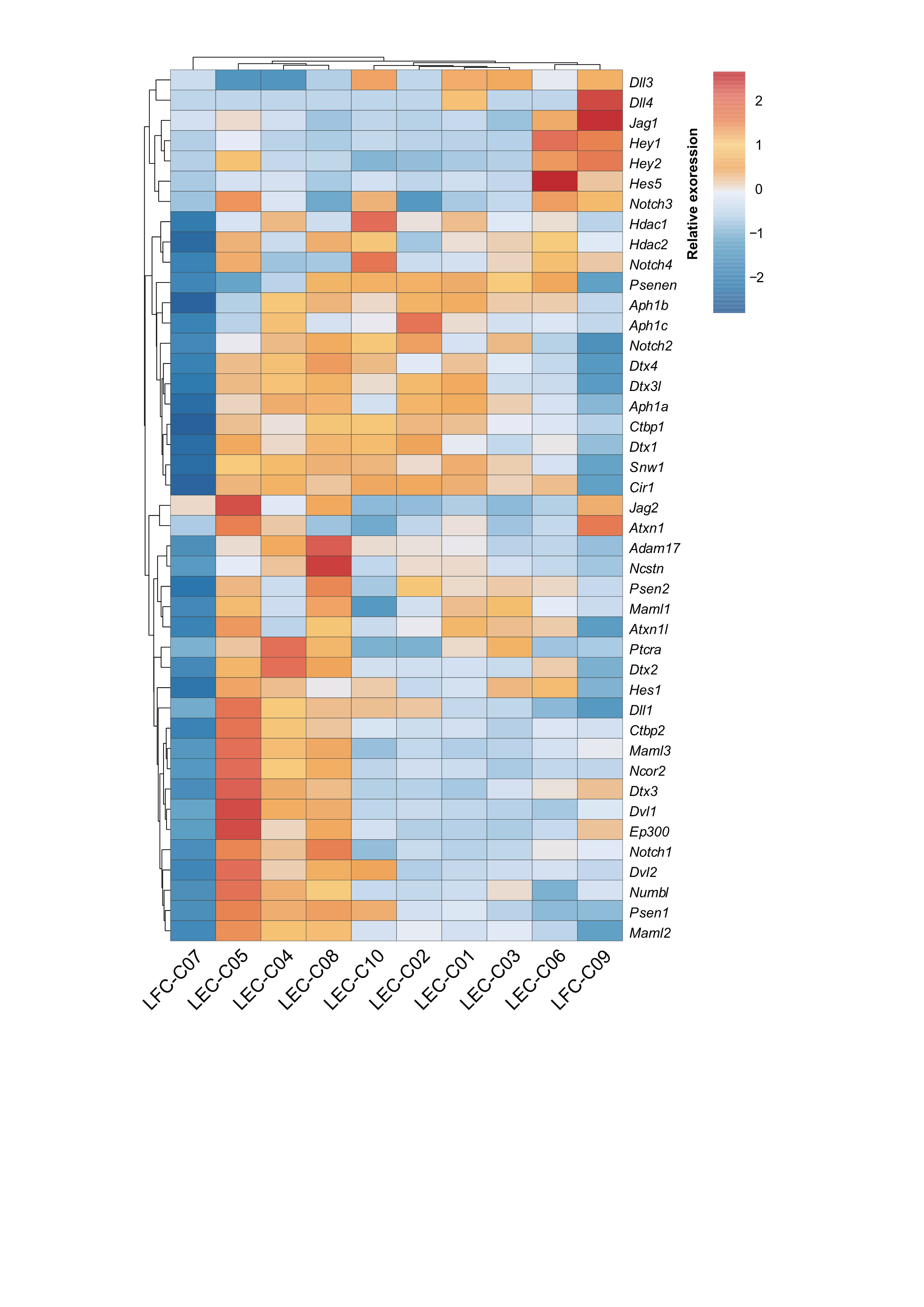

Supplement: Supplementary file 4 — Supplementary Figure S3. Heat map showing the expression patterns of all genes related to Notch signalling pathway in LEC clusters. [file CPR-56-e13412-s001.tif]

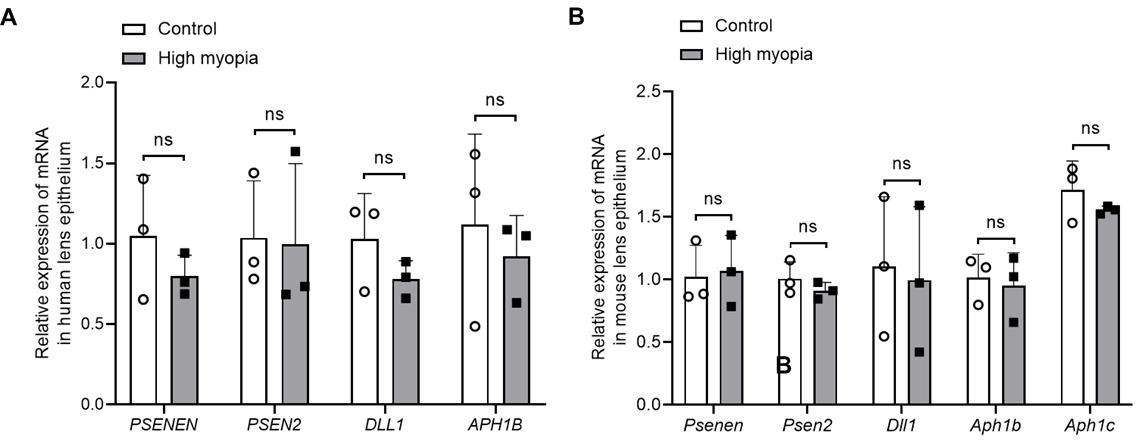

Supplement: Supplementary file 5 — Supplementary Figure S4. The mRNA levels of genes related to NOTCH signalling revealed by pseudotime analysis in human and mouse highly myopic lens epithelium. ns, not statistically significant. [file CPR-56-e13412-s006.jpg]

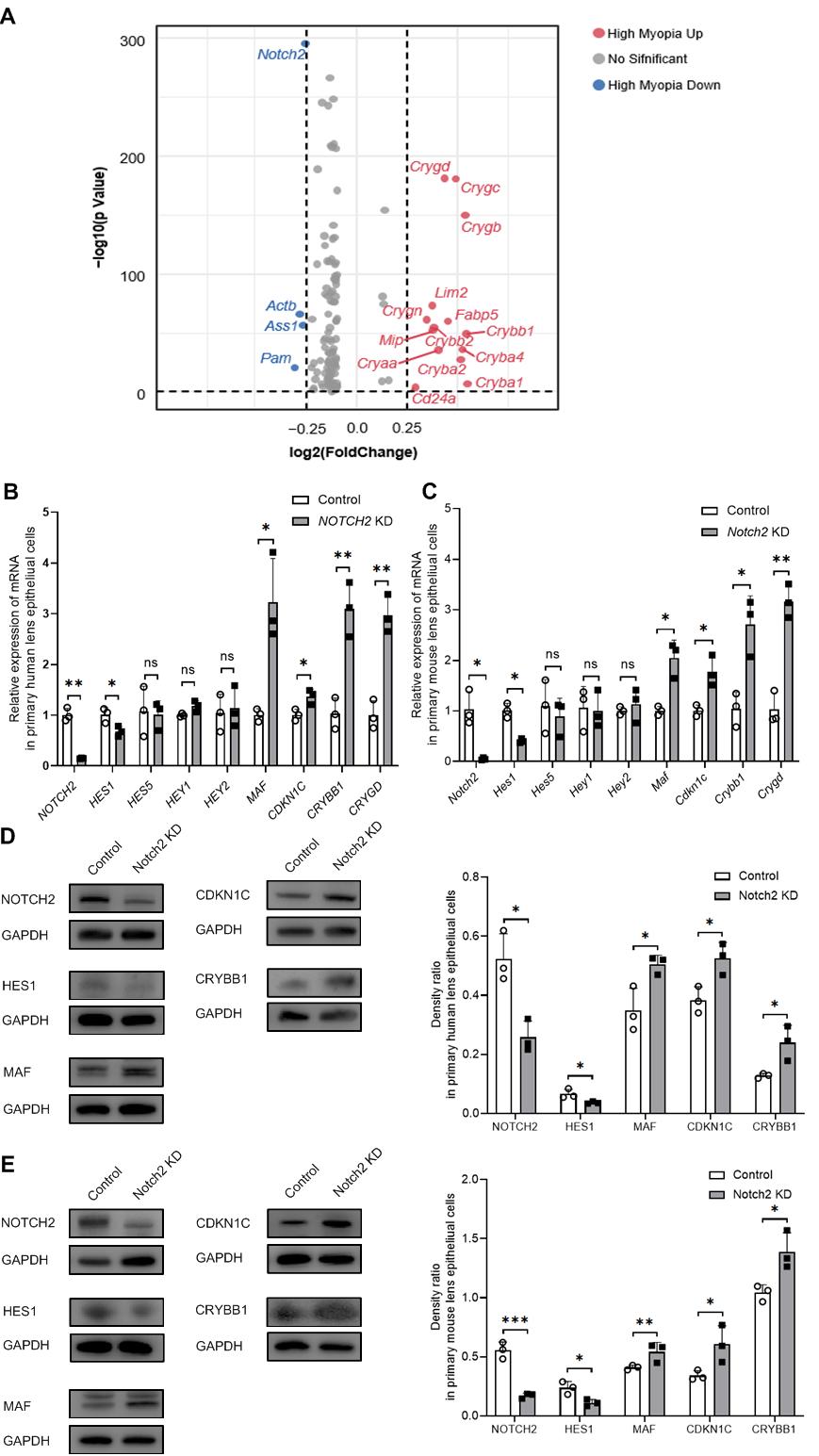

Supplement: Supplementary file 6 — Supplementary Figure S5. (A) Volcano plot displaying differentially expressed genes (DEGs) detected between the highly myopic and control lens epithelium. (B) The mRNA levels of downstream molecules in primary cultured human lens epithelial cells in response to NOTCH2 knockdown with siRNA. (C) The mRNA levels of downstream molecules in primary cultured mouse lens epithelial cells in response to Notch2 knockdown with siRNA. *p < 0.05; **p < 0.01; Student's t‐test. (D) Western blotting analysis of downstream molecules in primary cultured human lens epithelial cells in response to NOTCH2 knockdown with siRNA targeting another region of NOTCH2. (E) Western blotting analysis of downstream molecules in primary cultured mouse lens epithelial cells in response to Notch2 knockdown with siRNA targeting another region of Notch2. Following NOTCH2 knockdown, its effector HES1 was significantly down‐regulated, while other downstream molecules, Hes5, Hey1, and Hey2 were not significantly changed, accompanied by the significant up‐regulation of MAF, CDKN1C, and fibre cell markers (CRYBB1 and CRYG) in both human and mouse primary lens epithelial cells. Data are expressed as mean ± SEM. *p < 0.05; **p < 0.01; ***p < 0.001; ****p < 0.0001; Student's t‐test. [file CPR-56-e13412-s007.jpg]
