# Supplementary material for: Single‐cell RNA sequencing: Inhibited Notch2 signalling underlying the increased lens fibre cells differentiation in high myopia
Source: Cell Prolif. 2023 Jan 30;56(8):e13412. doi: 10.1111/cpr.13412 (PMC10392066; doi:10.1111/cpr.13412)
Supplement: Supplementary file 7 — Supplementary Table S1. Primer sequences for qPCR. Supplementary Table S2. Antibody list. [file CPR-56-e13412-s002.docx]

**Supplementary Table S1.** Primer sequences for qPCR.

| Gene | Genome | Forward Primer | Reverse Primer |
| --- | --- | --- | --- |
| *CRYBB1* | Homo sapiens | CAGCGTGAAGGTCTCCAGTG | GACTGCATCTGTGGCTGGAA |
| *CRYGD* | Homo sapiens | CCTGTCTTCAGGACCGCTTC | CCCTGGCATCAGCAGGTACT |
| *PSENEN* | Homo sapiens | CTGGAGCGAGTGTCCAATGAG | GCGCCAGACATAGCCTTTGAT |
| *PSEN2* | Homo sapiens | AGTGTGTGATGAGCGGACG | ACTGGGCAGTGTTCTCTCCAT |
| *APH1B* | Homo sapiens | CGAGCCGTTGCGTATCATCTT | CCAAACAAGGGACGAAATCAGT |
| *DLL1* | Homo sapiens | GATTCTCCTGATGACCTCGCA | TCCGTAGTAGTGTTCGTCACA |
| *MAF* | Homo sapiens | CAGCAAGGAGGAGGTGATCC | GCTGGTTCTTCTCCGACTCC |
| *CDKN1C* | Homo sapiens | GCGGCGATCAAGAAGCTGT | GCTTGGCGAAGAAATCGGAGA |
| *HES1* | Homo sapiens | TCAACACGACACCGGATAAAC | GCCGCGAGCTATCTTTCTTCA |
| *HES5* | Homo sapiens | GAAAAACCGACTGCGGAAGC | GACGAAGGCTTTGCTGTGCT |
| *HEY1* | Homo sapiens | GTTCGGCTCTAGGTTCCATGT | CGTCGGCGCTTCTCAATTATTC |
| *HEY2* | Homo sapiens | AAGGCGTCGGGATCGGATAA | AGAGCGTGTGCGTCAAAGTAG |
| *GAPDH* | Homo sapiens | GAAGGTGAAGGTCGGAGTC | GAAGATGGTGATGGGATTTC |
| *Crybb1* | Mus musculus | GTCTTCGAGCAGGAAAACTTT C | CTTCTCCAGGACAAACATCTC T |
| *Crygd* | Mus musculus | GATGGGTTTCAGTGACTCTGT C | TATCATCTGGCCTCTGTACTC T |
| *Psenen* | Mus musculus | ATGAACTTGGAGCGGGTATCC | CGAGGAACGCCTCTCTGAAG |
| *Psen2* | Mus musculus | GTATGGGGCGAAGCATGTGAT | ACGCACAGACTTGATAGTGGC |
| *Aph1b* | Mus musculus | TGGTCGTCATAATGCTGCACG | CACCAGATGCGTCAGGAGA |
| *Aph1c* | Mus musculus | CCTGTGTTCTTCGGTTGCG | CATGGACGAAAGCAGGAGAGA |
| *Dll1* | Mus musculus | GCAGGACCTTCTTTCGCGTAT | AAGGGGAATCGGATGGGGTT |
| *Maf* | Mus musculus | GCACTTCGACGACCGCTTCTC | TCGGATCACCTCCTCCTTGCTG |
| *Cdkn1c* | Mus musculus | CGAGGAGCAGGACGAGAATC | GAAGAAGTCGTTCGCATTGGC |
| *Hes1* | Mus musculus | TCAGCGAGTGCATGAACGAG | CATGGCGTTGATCTGGGTCA |
| *Hes5* | Mus musculus | AGTCCCAAGGAGAAAAACCGA | GCTGTGTTTCAGGTAGCTGAC |
| *Hey1* | Mus musculus | GCGCGGACGAGAATGGAAA | TCAGGTGATCCACAGTCATCTG |
| *Hey2* | Mus musculus | AAGCGCCCTTGTGAGGAAAC | GGTAGTTGTCGGTGAATTGGAC |
| *Gapdh* | Mus musculus | CATCACTGCCACCCAGAAGACTG | ATGCCAGTGAGCTTCCCGTTCAG |

**Supplementary Table S2.** Antibody list.

| Protein | Brand | Catalog number | Dilution |
| --- | --- | --- | --- |
| CRYBB1 | Santa Cruz | [sc-48335](https://www.scbt.com/zh/p/betab1-crystallin-antibody-h-3?requestFrom=search) | 1:100 |
| CRYG | Santa Cruz | [sc-365256](https://www.scbt.com/zh/p/gamma-crystallin-antibody-b-5?requestFrom=search) | 1:100 |
| NOTCH2 | Cell Signaling Technology | 5732 | 1:1000 |
| NOTCH2 | Sigma Aldrich | ZRB1830 | 1:100 for IHC |
| HES1 | Abcam | ab108937 | 1:1000 |
| CDKN1C | proteintech | 66794-1-Ig | 1:1000 |
| MAF | proteintech | 55013-1-AP | 1:1000 |
| GAPDH | Cell Signaling Technology | 5174 | 1:1000 |
